# ‘The Advice of a Gent Who Died from Neglecting it’: The Gentlemanly Pursuit of Knowledge Regarding Domestic Medicine in Kent c.1630−1800

**DOI:** 10.1093/shm/hkae041

**Published:** 2024-10-08

**Authors:** Francesca Elizabeth Richards

**Keywords:** domestic medicine, gender, reading, knowledge, networks

## Abstract

English gentlemen in the early modern period held ultimate responsibility for the health of their households. Building on previous studies which have revealed how both men and women of the gentry participated in remedy-collecting and some forms of caring duties as necessity demanded, this article situates gentlemanly interest in domestic medicine within familial, social and professional networks of knowledge and reading practices. Employing a micro-historical approach, this study explores the interests of Sir Henry Oxinden of Barham and his great-grandson, Lee Warly of Canterbury, who developed their medical knowledge by consulting female relatives, local acquaintances and medical texts. They assessed the value of physicians’ advice and the appeal of new ingredients. This article thus contributes a significant case study to the historiography of domestic medicine, presenting the gentlemanly pursuit of medical knowledge for practical and academic purposes as an activity which enhanced male status within the family and community.

While men and women’s interest in health and domestic medicine during the early modern period has been well-documented in recent scholarship, the historiography lacks detailed case studies which explore how lay gentlemen developed their medical knowledge to benefit themselves and their families.^1^ How did gentlemen of the early modern period gain knowledge about medicine and whom did they trust as experts in sickness and health? The letters, notes and annotated medical books of Sir Henry Oxinden (1609−1670) and his great-grandson, Lee Warly (1715−1805), offer the opportunity to examine the interests of early modern gentlemen in domestic medicine. Comparing the worlds of Oxinden, a seventeenth-century country squire and Warly, an eighteenth-century bachelor attorney, presents fascinating insights into how they gained knowledge, whom they trusted and how they responded to new medical developments.

This study builds on previous scholarship on early modern domestic medicine, gender relations and reading practices. Women have been portrayed as the altruists of domestic medicine with studies of gentlewomen’s diaries, letters and receipt books revealing the skills and knowledge involved in recording and manufacturing home remedies and providing healthcare both to their households and local communities.^2^ While Gervase Markham’s reference to ‘physick’ as the primary duty of *The English Hus-wife* (1615) has reinforced the notion that domestic medicine was limited by gender, research into gentlemen’s diaries and correspondence reveals that men too took a keen interest in health and medicine at home.^3^ In Roy Porter’s classic essay, ‘The Patient’s View’, his discussion of two well-known seventeenth-century gentlemen, Samuel Pepys (1633−1703) and Ralph Josselin (1616−1683), highlights the deeply-felt daily concern about health in a period of intense *ill* health including bouts of plague and high child mortality.^4^ Their diaries reveal their lived experience both as patients and concerned relatives and Porter argues that they were active medical researchers, seeking advice within their social network and a diverse medical marketplace, where consulting physicians was an option rather than the rule.

More recently, historians of early modern medicine including Lisa Smith, Hannah Newton, Jennifer Evans and Sara Read have explored further the care and concern shown by gentlemen towards sick members of their household, particularly their wives and children.^5^ Smith argues that safeguarding the health of his family was a fundamental responsibility of the patriarchal head of the household, involving administering medications, tracking intimate details of a relative’s condition or consulting others for advice. As husbands, fathers, brothers and sons, men were personally affected by illness within the family and saw it as their moral duty to care for their dependants, especially when the women of the family were emotionally or physically unable to provide care themselves. Men also had an obligation to maintain their own health; without the head of the household to manage the finances and bring in income, life would be very difficult for his dependents.^6^

Alun Withey, Elaine Leong and Sara Pennell have established the role of men in collecting, adapting and documenting domestic remedies. Men, as well as women, exchanged remedies within social networks and garnered knowledge about ‘what and who was trustworthy’.^7^ Gentlemen in Wales recorded medical receipts they collected in diverse places, from commonplace books to account ledgers, farming tithe records and books of legal precedents.^8^ In contrast, the Fairfax ‘family books’ reveal collaboration between men and women in compiling precious books of family remedies, intended to transmit knowledge down generations.^9^ These studies suggest that, despite popular discourses regarding gender roles and spheres of responsibility, gentlemen as well as gentlewomen, took an active interest in domestic medicine, in a practical sense as well as an academic one.

Sir Henry Oxinden and Lee Warly were both avid letter writers and book collectors whose notes and correspondence demonstrate a curiosity about medicine, considering practical remedies and the finer details of wider medical discourses. Their papers and book collections thus offer a particularly rich source of material to further explore gentlemanly attitudes to health. Oxinden’s commonplace book resides in the Folger Shakespeare Library and the family letters were published by Dorothy Gardiner.^10^ Oxinden’s book collection was passed down via his daughter Katherine and grandson John to Lee Warly, where it was incorporated into Warly’s extensive collection.^11^ Some of the Warly correspondence is held at the British Library and the University of Reading. There are 32 medical books in the collection, which were exhaustively analysed for annotations for this study, as well as other volumes which reveal broader cultural attitudes relating to health. Collating annotations, family correspondence and personal notes provides a broader understanding of their attitudes towards domestic medicine and the study and sharing of knowledge within this Kent gentry family.^12^

Exploring these gentlemen’s engagement with medical texts can be situated within extensive scholarship on early modern reading practices. Peter Murray Jones’ case study in Tudor Cambridge highlights the ‘lay culture of medicine’ in which non-professionals owned a broad range of medical books and shared a culture and language of medicine with physicians.^13^ However, as he states, ‘owning a book is not the same as reading it’ and the inscriptions in the books owned by the Oxindens and Warlys are significant as evidence they studied their medical books. Kevin Sharpe and Steven Zwicker note that marginalia draws our attention to the ‘precise moments in which meanings are historically formulated.’^14^ Thus, rather than taking medical texts as ‘stable’ and uncontested, we can explore how they were understood by Oxinden and Warly as members of different ‘interpretive communities’. We can also compare the aims of Oxinden and Warly in the light of research by Anthony Grafton, Lisa Jardine, William Sherman and Ann Blair who argue that the study of texts can be interpreted beyond general knowledge accumulation; note taking was orientated towards achieving a specific practical goal, for exchange in a ‘knowledge transaction’ or distilling information perceived to be important for future generations.^15^ While Zwicker suggests there is an overarching decline in active engagement with printed material from the turbulent period of the civil wars and Restoration to the more stable eighteenth century, the reading and note-taking practices of Oxinden and Warly regarding health nuance this grand narrative.^16^

Karen Harvey has highlighted the discomfort which comes from returning the gaze to men and masculinity, after hard-fought efforts to bring women’s history into the limelight, but argues that multiple versions of masculinity throughout the early modern period require attention.^17^ Alexandra Shepard notes that the history of gender requires further analysis of the ‘multirelational context’ of masculinity and ‘conflict… within each sex as well as between them.’^18^ This study situates male interests and motivations within the context of their family relationships, local networks and the practical realities of their lives. This may present a more complex view of relationships with women and other men of similar social standing and a more sympathetic assessment of the responsibilities that accompany patriarchal power and privilege. Nevertheless, comparing the extensive notes of these gentlemen with the apparent absence of notes from women of the household throws into stark relief their differential legacy in the historical record, particularly in view of evidence that contemporary gentry women were engaged in the active study of medical texts.^19^

This study analyses how Oxinden and Warly understood health, whose advice they valued or scorned and how they sought and recorded relevant information. The first section introduces the key protagonists, situating their interest in medicine within their family circumstances, their reading practices and the broader medical landscape. The second section examines how Oxinden and Warly accessed medical information via familial, social and professional networks, evaluating the advice of relatives, local acquaintances, writers and physicians on the basis of trust and respect. The final section explores how the critical study of medical texts emerges as a learned and gentlemanly pursuit, delving into their interest in specific medical conditions and their approaches to new imported ingredients for both practical and academic purposes.

Comparing a seventeenth-century gentleman and an eighteenth-century gentleman highlights the changing medical landscape and developments in accessing and interpreting information. However, Shepard emphasises the problems with comparing men of different classes, ages and circumstances, inevitably locating distinctions rather than continuities.^20^

This micro-historical approach focuses on how the individual situations of these two Kent gentlemen influenced their motivations in interacting with family members, networks and printed books to develop their medical knowledge and put it to use as an essential component of their familial duties. This study also asserts that despite differences in their needs and interests, these early modern gentlemen perceived both the *study* and *practice* of domestic medicine to fall within their remit of responsibility and expertise and sought to acquire knowledge at home and in the wider community.

## Sir Henry Oxinden and Lee Warly

Henry Oxinden could be described as the definitive seventeenth-century patriarch; a country squire, his rule extended to multiple households, wielding financial control over his mother and younger adult siblings as well as his wife, five children and servants. While acknowledging his authority, we should recognise the weight of responsibility which rested on his shoulders, particularly in the face of financial struggles, the civil wars (1642−1651), family illness and the challenges of their rural location. Oxinden’s first wife, Anne Peyton (1613−1640), suffered from consumption and died 8 years after their marriage; Oxinden then married his young ward, Katherine Culling (1624−1698), in 1642.^21^ Oxinden was injured during his one encounter as a Parliamentarian soldier, affecting his long-term health. Oxinden often turned to his aunt, Lady Margaret Oxinden, for advice on health matters and was part of a network of acquaintances who provided remedies: Oxinden recorded 40 medical receipts in his commonplace book, his *Miscellany*, to treat coughs, colds, toothache, sunburn and consumption.^22^

Oxinden owned seven medical books including two describing specific ingredients: Culpeper’s *A Physical Directory* (2nd edn, 1650) detailing drugs in the College of Physicians’ *Pharmacopeia* and a lapidary, Thomas Nicols’ *Gemmarius Fidelis* (1659).^23^ He also owned a household management manual translated from the French, Charles Estienne’s *The Countrey Farme* (1616), which includes remedies and discourse on specific foreign ingredients and two anatomy books, Thomas Vicary’s *The English Man’s Treasure* (1613) and Helkiah Crooke’s *Mikrokosmographia* (2nd edn, 1631).^24^ Henry’s friend, Marchamont Nedham, gave him a copy of his book, *Medela Medicinae* (1665).^25^ Oxinden also annotated a cosmography and a book on ‘marriage duties’ and owned a copy of Francis Bacon’s *The Essays, Or Counsels Civill and Morall* (1639).^26^ When exploring Oxinden’s interest in health and remedies, we can interpret his reading and note-taking practices as ‘goal-orientated’, his library supplemented by advice and information from relatives and acquaintances in addressing the key health concerns of his family.^27^

In contrast, Henry Oxinden’s great-grandson, Lee Warly, was a wealthy man with few family responsibilities and time to indulge his interests. He was an only child, devoted to his widowed mother, Mary Warly née Lee (1682−1769). While interested in medicine, Lee Warly chose not to follow in the footsteps of his father John Warly (d.1732), a surgeon, and was admitted as an attorney to the Courts of King’s Bench and Chancery in 1736. He bought a large townhouse in Canterbury High Street in 1741, living there with his mother until her death and then alone but attended by three servants.^28^ As a lifelong urban bachelor, Warly’s privileges as a wealthy, educated man within a patriarchal system allowed him a large degree of freedom and his close relationship with his mother was one of good humour and care. He could be understood as a ‘polite gentleman’ of the eighteenth century, in which the performance of masculinity was associated with learning, civilised behaviour and overt consumption of goods in an urban context, rather than asserting control within a household.^29^ Shopping, whether purchasing medicines at apothecary shops or books for his collection, was considered a sophisticated, sociable activity.^30^

Lee Warly inherited books on varied subjects from both the Warly and Lee families and made additional purchases. His medical library included Oxinden’s medical books and 18 of his father’s medical texts which are not annotated, likely bought for reference in his professional role as a surgeon. Two books combining cookery and health, *A Treatise of Foods* (1704) and *The Compleat Housewife* (16th edn, 1758) were owned by Mary Warly; Lee Warly inherited them on her death in 1769.^31^ Lee Warly likely cross-referenced between books he inherited and new purchases, *A Medical Dispensatory* (1657), *A Physical Dictionary* (1657), *The Treasury of Drugs Unlocked* (1690) and *The Vermin Killer* (1754) for detailed information about specific ailments and substances with perceived medicinal properties.^32^ Warly also bought *The Anatomy of Melancholy* (8th edn, 1676) by Robert Burton.^33^ Some of these choices indicate Warly filling in gaps in Oxinden’s collection.^34^ Oxinden did not own Culpeper’s *The English Physician*, published in 1652 and at the height of popularity during Oxinden’s lifetime, but Warly bought the enlarged edition, printed in 1698 after Oxinden’s death, to add to the family library.^35^ He also bought Blagrave’s supplement to *The English Physician* (1674).^36^ Warly’s copy of *A World Display’d* (1740), a satirical book on several stock characters of the period, presents evidence of his opinion on health practitioners.^37^ His copy of William Derham’s *Physico-Theology* (1742), a book of sermons, also gives an insight into the religious debates which influenced views of medicine.^38^ During this period of increasingly cheap print, Warly amassed a collection of 1,500 books which he studied with great verve and his extensive notes and annotations, cross-referencing and copying, demonstrate a coherent, active ‘project’.^39^

While Oxinden and Warly occupied very different worlds, there were enduring popular practices and understandings of health during this period. Both Oxinden and Warly were well-schooled in humoral theory which, since its development by Hippocrates and Galen, remained the fundamental framework for interpreting the human body. Imbalances of the four humours (blood, phlegm, yellow bile and black bile) were treated using procedures such as blood-letting or combining *materia medica*, known as ‘simples’, in compound medicines to achieve the desired results for the individual’s constitution. During the seventeenth century, challenges to this traditional model came from proponents of chemical medicine. These physicians sought to develop pure chemical preparations for universal application and some adopted a particle-based view of medicine and physiology according to the tenets of corpuscular philosophy.^40^ However, balancing the four humours remained a widely accepted way of understanding health throughout the early modern period.^41^ Domestic medicine continued to constitute the mainstay of treatment, while prolific medical literature in print provided access to new remedies and medical debates to a broad audience in the form of books, pamphlets and magazines.^42^ Scholars have recognised the high level of medical knowledge amongst lay men and women, noting a ‘common, open, intellectual culture’.^43^ Leong and Pennell suggest this knowledge was central to being an ‘able participant’ in a ‘mixed medical economy’ of apothecaries, physicians, empirics and healers.^44^ Thus, Oxinden and Warly would have understood their own expertise in health as both valid and essential within the medical landscape of early modern England.

Oxinden and Warly would also have conceptualised health in religious terms, recognising a moral duty of care for their own bodies and souls and those of their dependents. Oxinden and Warly were both committed Protestants; Oxinden became an Anglican vicar later in life and the Warlys owned popular religious texts on piety. A Protestant view of health included adopting sensible, modest lifestyle habits, living in a moderate, temperate climate and using suitable medicines to relieve symptoms.^45^ Moderation also extended to the moral economy of the household; an emphasis on frugality and thrift throughout this period demanded that purchases, even medical purchases, were justified as necessary expenses.^46^ However, ultimately the survival of the patient depended on the will of God.

## Knowledge Networks

As heads of the household, both Oxinden and Warly were responsible for ensuring their dependents’ health: recording information, accessing help and advice and obtaining medicines. Oxinden was practically engaged with family health on a quotidian basis, as his family suffered regular bouts of sickness. Letters to and from Oxinden mention illnesses ranging from colds, coughs, sore wrists and ‘cholick’ to measles, smallpox and consumption.^47^ Given the well-established role of gentry women in domestic medicine, we might assume that Oxinden’s wife would take on primary responsibility for healthcare. Yet, as Oxinden’s first wife Anne was frequently unwell and his second, Katherine, had become a wife and step-mother to three children at the tender age of 18, Oxinden relied on his aunt, Lady Margaret Oxinden of Dean, as an established medical authority in the family. Margaret often wrote to Oxinden about family health and visited Great Maydekin to nurse sick relatives. When Katherine was struck down with measles, Oxinden writing on 26 May 1646, credited Margaret with her recovery, ‘The Lady Oxinden and the Ladie Percival next under God restored her to that health which His almighty selfe first gave her’.^48^

Yet when Margaret could not attend herself, it fell on Oxinden to take charge of his sick wife. This reflects Protestant discourses on marriage during the period which stated that husbands and wives should care for each other’s physical, emotional and spiritual health.^49^ Oxinden wrote that Anne was ‘verie ill of an extreame cold’ on 13 January 1637.^50^ Margaret sought to reassure Oxinden that the self-help strategies he employed would help, ‘I hop not dayngerus, with such good meanes as you use, ther for I pray do not be dismayed.’^51^ Though it is unlikely that Oxinden provided nursing care or made medicines himself, his letter suggests that directing Anne’s care and managing the manufacture and administration of medications fell on Oxinden’s shoulders. Upon Anne’s illness in 1640, Margaret wrote to Oxinden: ‘I will make her a tisain to morrow and send her to eas the paynfullnes of her coff, which you shall have sent you by to or thre of the clok’.^52^ The same year, Margaret sent medicines for Oxinden’s mother, Katherine, along with full instructions on administration:

I send her allso a powder which I wold have her take in a litel beer or posit, which she likes best, as much as will ly a pon 3d. will be enuf at a time, that or the water may be taken at any tim when she is ill. She may take this water with heat as other hot water is takin, so wishing her health and you al happines, I rest

Your afectionat frend and Ant

MARG: OXINDEN.^53^

These letters indicate that Margaret wrote directly to Oxinden, a concerned husband, father and son, advising him on appropriate medicines and dosages and also providing him with emotional comfort in times of worry and strain.

Warly never married and had no children so his interest in healthcare might be considered more academic. However, like Oxinden, Lee Warly was also responsible for this mother’s welfare. While up in London, Warly wrote to Mary to inform her that he had purchased medicines for her known as ‘sugars’ and was sending them down by sailing vessel.^54^ While Oxinden relied on his aunt Margaret to send medicines or instructed servants to make medicines according to receipts, Warly could purchase proprietary medicines from the increasing array of apothecary shops in London. The main area for apothecary shops was Cheapside, near the Courts where Warly practised as an attorney.^55^

While the exchange of letters between Oxinden and his aunt Margaret frequently expressed anxiety, desperation and grief, the Warlys seem to have enjoyed better health overall and treating ailments could even be a topic of humour. Mary wrote to Warly in May 1739, recounting an amusing attempt by an ‘aunt’ to provide care:

Mrs Six entertained us with a story of Franck Wraith which set us in a great fit of laughter … He had got a great cold and a very stiff neck so that he could not turn his head about. Her aunt… bathed it which she thought proper, for about an hour. She ask him if he found good by it, he answers, Indeed forsooth I don’t know, you have been rubbing the wrong side.^56^

This anecdote demonstrates that medical topics presented a source of conversation and amusement between Warly and his mother. In this case, the aunt in question was presented as lacking in medical knowledge, rather than a source of expertise. Family correspondence highlights the contrasting circumstances of Oxinden and Warly; while Oxinden sought advice to protect the lives of his nearest and dearest, Warly could enjoy a more relaxed approach to health, enjoying a joke and having the money and opportunity to buy sweet, restorative medicines for his beloved mother.

Acquiring medical information through wider local networks could be stimulating and often sociable. Oxinden and Warly were part of a lively gentry community in Kent who shared a questioning, rational engagement with husbandry and health since the sixteenth century and as devout Protestants, the health of mind, body and household was a spiritual as well as a practical concern.^57^ There were many gentry authors writing on health and husbandry in the local area: the Digges of Chilham, the Culpepers of Wigswell and the Scots of Scot Hall. The prolific writer and translator Gervase Markham lived for a time with his patrons, the Sidneys, just over the border in East Sussex.^58^ The Oxindens were likely acquainted with some of these writers. Oxinden records lending a book to ‘Mr Diges’ in 1656.^59^

This network offered not only intellectual stimulation but also practical advice. 26 receipts out of 40 recorded in Oxinden’s *Miscellany* are attributed to 20 named contributors, from local gentry, clergymen, clerks and friends to acquaintances.^60^ These included John Payne, a Clerk of the Chancery, Henry Birkhead, a Latin poet, Sir Robert Crasford, possibly a local squire, John Swan, Rector of Denton and ‘old Mr Vincent’, presumably Oxinden’s long-standing friend, Vincent Denne.^61^ Only one receipt is credited to a woman, Miss Weedon and only two physicians are listed directly, Dr Hawtin and Charles Annott. We can conceptualise these snippets of information as ‘gift medicine’, a term used by Anne Stobart.^62^ She refers to the exchange of medical receipts between gentry families which took place primarily between women to enhance status and prestige. We do not know whether Oxinden offered reciprocal receipts but it was usually men of equal or lower status providing these remedies.

The noting down of remedies for common ailments in the Oxinden family suggests practical and immediate use. There are 33 recipes for coughs and colds which the family was frequently afflicted with. Anne Oxinden died from consumption in 1640; Oxinden’s inclusion of the following receipt was possibly an attempt to safeguard against a similar fate for another relative.

Against a consumption

Take three pints of the best Canaly, halfe a pound of loafe sugar, three nut megs pricked with needles, & put them in a bottle, and let them stand three weekes, then take a quarter of a pinte in the morning, with the yoalks of two new layd eggs. Mr Rauger of Douer May. 1658.^63^

The treatment was credited to Mr Rauger of the nearby port of Dover, the result of a verbal or written exchange or received second-hand. Withey notes that recipes ‘moved organically through networks, regions and peoples’ and remedies ‘transcended barriers of class, geography, literacy’ and even ‘language’.^64^

The social networks of Henry Oxinden reflect what Ilana Ben-Amos calls the ‘two major clusters’ of ‘reciprocities’ which overlapped and intertwined: that is one of ‘kinship, neighbourly and friendship ties’ that ‘involved relations built on trust’ and a sense of equal exchange and one which revolved around systems of patronage.^65^ Rather than being in the ‘powerful and privileged position of the giver’, Oxinden’s notes and letters reveal the remedies he received from relatives and acquaintances of equal or lower standing which may suggest that men and women shared recipes with him both as a form of equal exchange and to assert their position within the community, with recipes acting as a form of ‘social capital’.^66^

Lee Warly’s family was well-established in Canterbury and part of extensive local networks. His maternal grandfather was a Canterbury alderman.^67^ His father, ‘John Worley’, is listed as a Surgeon of Canterbury, made a Freeman in 1708 and active c.1710.^68^ As Lee Warly worked as an attorney in London and was an enthusiastic book collector, it is likely he was well known within the legal world and had contacts among London booksellers, in addition to the social circles of his family and acquaintances. There is little evidence that Warly collected remedy receipts from social contacts like Oxinden. This may be explained by his much smaller household or the increasing availability of proprietary medicines during the eighteenth century, not least sold by booksellers.^69^ Warly lived an urban life with few dependants and his networks may be understood as more scholarly.

However, as Oxinden’s social networks reveal dimensions of class, gender and profession, Warly’s extensive book-collecting practices assert him as part of a community of wealthy professionals who bought books in London. Contemporaries of Warly collected diverse books: Thomas Baynham, a barrister of the Middle Temple, died in 1783 leaving a collection of over 6,000 books, only 7 per cent of which were legal texts.^70^ Eighteenth-century London booksellers arranged books by genre such as ‘Physick, Surgery etc’ and social status so that the choice and purchase of a book asserted the class identity of the purchaser.^71^ However, Warly was not simply purchasing medical books as an act of overt consumption. His cover-to-cover annotations reveal engagement with the texts and he may have discussed key medical texts with knowledgeable booksellers and fellow book enthusiasts.

During the early modern period, the idea of ‘every man his own doctor’ was prevalent, based on the everyday accumulation of medical knowledge amongst people of all classes and the increasing availability of medical texts.^72^ While the College of Physicians sought to assert their authority, the populace was well used to treating themselves or consulting a wide diversity of practitioners, depending on their perceived expertise and the price. Oxinden and Warly were both very capable of gathering information on the causes and treatments of common health conditions and they both assessed the merit of consulting physicians.

The Oxindens counted several physicians within their social network and Oxinden was personally acquainted with Marchamont Nedham (1620−1678), journalist, medic and author of *Medela Medicinae* (1665).^73^ Oxinden’s copy of this book has the names of both Nedham and Oxinden in it and the inscription ‘*Ex dono authoris* Xmas day 1664’ indicating the book was a gift. Oxinden also lent physicians learned books on physic: in 1657 he recorded lending a manuscript of physic, a Spanish physick book and a Dioscórides to Mr Jacob, likely Dr William Jacob, a physician of Canterbury actively practising medicine c.1649−1687.^74^ These texts may have been in Latin and required an educated readership, beyond that of the popular medical books in the vernacular widely available to any literate person to buy. This loan indicates the two-way flow of knowledge between this educated gentleman and physicians.^75^

From the 20 contributors credited in Oxinden’s *Miscellany*, only three are identified as physicians and receipts credited to them are not given greater weight than receipts from lay acquaintances. Oxinden wrote, ‘An excellent Pouder for the tooth, as Charls Annootes affirmees’.^76^ Charles Annott, physician and surgeon of Canterbury, was attendant on the girls’ school where Oxinden’s daughter resided.^77^ For a cough, ‘this is Doctor fox his receipt teste Mr Parker’, likely Dr William Fox, surgeon of Canterbury, who was married to Annott’s daughter.^78^ These medical recommendations were gleaned via informal kinship networks, rather than professional consultations.

Oxinden’s attitude to physicians reflects the open medical culture of the period. He owned medical texts on anatomy, diseases and cures. He compiled useful receipts to manage domestic medical concerns himself and could rely on the expertise of his aunt, Lady Margaret, to cope with household emergencies. Even when the Oxindens sought the help of a physician, a licensed practitioner might be unable to attend or offered little additional expertise. When Anne caught a severe cold in the snow in 1637, Oxinden sent for Canterbury physician, Dr Edmund Randolph. Randolph held qualifications from Oxford and Padua, but his attendance was dependent on travelling in poor weather conditions.^79^ A doctor was called again to Anne in 1640 but without success and Margaret had little faith that it would help: ‘I am very sorry to hear that my Neece is so ill still, but shuch is the Nature of this kind of sikneses that I am veryly perswayd it is not in the powr of any phisition to alter’.^80^

While Oxinden respected local physicians as educated, upstanding men like himself, the actions of the College of Physicians may have harmed his trust in that professional body. While ‘a physician of good character, who could exercise good judgment and advise a man of learning’ was valued, the College’s efforts to control licensing, prosecute irregular practitioners and condemn attempts to make medicine more accessible to the masses undermined the moral element of the profession.^81^ Oxinden’s choice of books might reflect this perception of the College. Oxinden bought Culpeper’s *A Physical Directory* (1650) which expressly sought to undermine the control and elitism of the College by translating the *Pharmacopoeia Londinensis* of 1618 from Latin into the vernacular. Oxinden also bought Crooke’s *Microkosmographia* (1631), again condemned by the College. Finally, Oxinden’s friendship with Marchamont Nedham is revealing as Nedham wrote in *Medela Medicinae* (1665), that he would rather consult an experienced and ‘prudent apothecary’ who observed the unique character of the patient than a physician who rigidly followed medical doctrine.^82^ Oxinden’s interest in chemical medicine and his friendship with Nedham, a signatory to the petition to establish a Society of Chemical Physicians, indicate an openness to new ideas.

Warly’s notes and annotations openly express distrust and derision regarding the medical profession. Lee Warly has inscribed a poem by Reverend John Warly, Henry Oxinden’s son-in-law and Lee Warly’s grandfather on the front pages of *A Treatise of Foods* (1704) and at the back of *The Anatomy of Melancholy* (1676):

All mischief seems far off when it is near

It ceases to be such caus’t doth appear;

It is Fate’s method to conceal decree,

What’s most pernicious, that man seldom sees,

When Nature fights, and doth resolve to kill,

She fools Physicians with pretended skill,

By what man fails those men of art scarce know,

Till Death strips man and shows the mortal blow.^83^

John Warly’s poem, printed in 1674, portrays the perceived ineptitude of physicians against Nature and is especially poignant given his own premature death, 5 years later. The physicians with their ‘pretended skill’ appear fraudulent, intent on tricking their doomed patient and the notion of ‘those men of art’ who ‘scarce know’ negatively contrasts a university medical degree with more hands-on experience.

Physicians could be viewed as being pompous and deliberately obscure, especially in a medically pluralist society where apothecaries, surgeons and lay-healers all challenged their expertise. As the son of a surgeon, Warly may have particularly resented physicians’ claims to superiority. The authors of the Warlys’ *A Physical Dictionary*, a diverse group of surgeons, apothecaries, students and others, wrote that the book was intended ‘for the help of charitable and honest-meaning people’.^84^ This contrasts with the unreliable, even misleading, physicians they describe below:

Sirs, here you will find the confused Recipes, and linsy-woolsy conceptions of Physicians ranged into an exact method, their Enigmatical expressions unforked and unveiled, their cloudy sentences artionobilised into rayes of Light.^85^

Physicians were also represented as wasteful, extravagant and immoral. In Warly’s copy of *The World Display’d* (1793), a satire on the characters of early modern society, he underlined the following description of ‘A meer dull Physician’:

He translates his Apothecary’s shop into your chamber and the very windows and benches must take physic…Noblemen use him for a director of their stomach and ladies for wantonness, especially if he be a proper man. ^86^

This caricature of the physician presents him as a man employed at the whim of the nobility to meet their various appetites, rather than a respected authority on health. Given the close link between moral and physical health posed by Protestant authors of the period, with a firm emphasis on moderation in all things, a corrupt and contemptible physician could no more cure the body than he could cure the soul.^87^

In the front of *A Treatise of Foods* (1704) and again at the back of *The Anatomy of Melancholy* (1676), Warly wrote an anecdote about ‘The celebrated Dr Boerhaave’, who apparently ordered all his books to be burnt upon his death, apart from ‘one large volume with gilt leaves and silver clasps’. When bought for ten thousand gilders by a German count, the pages inside were blank except for a note stating, ‘Keep the head cool, the feet warm and the body open: and bid defiance to the physician.’ Herman Boerhaave (1668−1738), the prominent Dutch physician at the University of Leiden, emphasised a focus on anatomy, practical observation and scientific investigation.^88^ Whether Warly engaged with Dr Boerhaave’s teachings or not, he seems to have enjoyed the spirit of rebellion in the anecdote.

Warly’s attitudes may be situated within a broader ambivalence towards physicians in the eighteenth century. While Porter argues that the popular periodical, *The Gentleman’s Magazine*, shows very little hostility towards health practitioners and a collaborative culture between professional and lay contributors, he also highlights the mocking depiction of physicians in cartoons and pamphlets.^89^ There was frustration during this period that, despite new medical theories and the availability of new medicines, there was little improvement in terms of efficacy.^90^ When Warly bought sugars for Mary in London, he was purchasing expensive medicines such as Syrup of Roses and Confection of Frankincense listed in the ‘Sugars’ section of Culpeper’s *The English Physician Enlarged*, a book which Warly added to Oxinden’s collection.^91^ Warly may have gone directly to an apothecary shop in London to purchase these medicines without consulting a physician, empowered by his home library to treat his mother himself.

## The Gentlemanly Study of Medicine

Within the Oxinden and Warly families, the study of medicine was very much a gentlemanly pursuit. Before considering the interests and motivations of Henry Oxinden and Lee Warly, it is worth exploring why this activity may have been restricted to the men of the family. There is evidence that some well-educated gentlewomen such as Elizabeth Freke and Margaret Boscawen read extensively, recording information and becoming formidable experts in health.^92^ Marks of female ownership in several books in the Oxindens’ and Warlys’ collection of domestic manuals, receipt books and medical books, references to female expertise in letters and examples of cooperation between men and women on health matters might also suggest that women such as Katherine Oxinden and Mary Warly had the opportunity to study medicine in the home library. However, this does not appear to have been the case.

There is very little evidence that Henry’s second wife, Katherine, studied domestic medicine or was even confident in writing. There seem to be no surviving letters and no book annotations in her hand. Her name appears in medical books but these were chosen by her husband to aid her role as a lady of the household. Oxinden gave his young wife two anatomy books and a general husbandry book. The choice of books reflects recommended reading material for women at the time; *The Gentlewoman’s Companion* (1678) suggested ‘several Books of Anatomie’, a herbal and general book of physic is required.^93^ Oxinden wanted Katherine to learn but within gender-defined limits. He underlined the following in *Marriage Duties* by Puritan theologian, Thomas Gataker:

A virtuous or industrious wife is the Crown of her husband… on the contrary, she is the contempt and dishonour of him when she striveth and contendeth to seem wiser than he. ^94^

This attitude reflects broader religious discourses about marriage in Protestant England, in which the power and wisdom of the patriarch as minister of the household was asserted and wives were the ‘junior partner’.^95^ Oxinden may have disapproved of Katherine writing in her books or she may have lacked the confidence to do so. Although Katherine had a boarding school education, Post-Reformation Protestant girls’ schools placed little value on learning to write and letters by other Oxinden women reveal embarrassment about their literacy skills, including apologies for ‘bad riting’.^96^ Heidi Brayman Hackel argues that ‘the scarcity of women’s marginalia’ has produced a ‘silence’ in the textual record, where female opinion was not expressed or required.^97^ While Katherine may have made informal notes elsewhere, now lost, the absence of her voice in family correspondence and her books is certainly striking.

Mary Warly’s name appears in *A Treatise on Foods* (1704) and *The Compleat Housewife* (1758), which contains many medical and culinary receipts. There are also 19 religious and educational texts in the name of Mary Warly or Mary Lee (her maiden name) in the Elham Collection. With improvements in female education by the eighteenth century, Mary wrote letters eloquently, yet there are few annotations in Mary’s hand in her books: a few examples of practising writing her name in her childhood books and one note recording the death of John Warly, ‘my husband’, in the flyleaf of *The true Church of England man’s companion in the closet* (1721).^98^ All annotations in *A Treatise on Foods* and *The Compleat Housewife*, including two recipes for pickles in the front flyleaf of the latter, appear to be in the hand of Lee Warly. The absence of notes by Mary is rather perplexing. Did she feel that annotations were unnecessary or could it relate to the fashionable style of feminine reading in the eighteenth century, reclining in a drawing room without access to a desk or quill?^99^ Her opinions may not be lost entirely. Some of Warly’s annotations might reflect the views of his mother, such as this comment communicating a female perspective on the character of the author in the preface of *The Compleat Housewife*: ‘the Good Housewifes say that Eliza Smith is an extravagant jade’. In the back of *The World Display’d* (1793), bought after Mary’s death, Warly has written ‘It is all for the best: MW’, perhaps endearingly quoting his mother. Nevertheless, the lack of female annotation in printed books belonging to women in the Oxinden and Warly families suggests it was considered a male prerogative.

Oxinden and Warly regarded the *study* of domestic medicine as a gentlemanly pursuit, appropriate to their sex, status and education. Both Oxinden and Warly, educated at Oxford or Cambridge, annotated their books and sprinkled their writing with Classical and scriptural references in Latin and English.^100^ Oxinden’s notes appear more intermittently and his use of a commonplace book suggests a more specific form of note-taking, while Warly’s extensive annotations throughout his books indicate he keenly read from cover to cover. The materiality of their books may have encouraged Oxinden and Warly to engage in this gentlemanly act of study in different ways. Oxinden’s books were often large, weighty tomes. Books such as *The Countrey Farme* (1616), *A Physical Directory* (1650) and *Cosmographie* (1657) have an air of gravitas and importance conveyed by their materiality. These books display a little underlining and a few small notes, as though too important to mar with handwritten musings. Oxinden may have made more notes in his commonplace book for this reason. By contrast, Warly’s books, often smaller in size and in cheap bindings, were filled with quotes and anecdotes and often objections, corrections and clarifications.

Studying medicine through perusing books and discussing issues of the day could have benefits beyond collecting practical remedies or accruing relevant medical information. Writing in the seventeenth century Francis Bacon, the philosopher and statesman, and Robert Burton, author and Fellow of Oxford University, both described study and discourse as essential to a healthy mind. In Bacon’s *The Essays* (1639), owned by Oxinden and well-annotated by Warly, Bacon wrote, ‘Reading maketh a full man; conference a ready man; and writing an exact man.’^101^ Warly also very heavily annotated *The Anatomy of Melancholy* (1676); he underlined in the conclusion, ‘Be not solitary, be not idle’.^102^ It is likely he perceived his use of his books and social networks as beneficial to his own health simply through the stimulation and engagement it brought him. Warly clearly enjoyed studying his extensive book collection and wished to be comfortable while doing so; in 1739, Warly wrote to Mary that he had bought an easy chair for his study and was sending it down from London.^103^

Oxinden and Warly also used their medical texts to research specific health issues. Oxinden was primarily concerned with chest complaints, both his own and his relatives’ and conditions relating to fertility and sexual health. As well as traditional Galenic medicine, he also listed chemical medicines such as brimstone (the vernacular term for sulfur) in his receipts and took an interest in theories gaining momentum in the seventeenth century such as corpuscular philosophy, the concept of moving particles invisible to the naked eye. In contrast, Warly tended to focus on personal lifestyle choices which affect digestion and melancholy, in accordance with Galenic notions of the six non-naturals pertaining to healthy habits such as sleep, diet and exercise and the four humoral constitutions: sanguineous, phlegmatic, melancholic and choleric.

Oxinden researched remedies within his books and noted brief summaries in his *Miscellany* for practical use. One of many cough remedies listed in Oxinden’s *Miscellany* could be derived from one in Vicary’s *The English Man’s Treasure* (1613):

Take brimstone beaten in powder halfe an ounce, and put it in a new laide egge soft roasted, mingle it well together, then put it into bengalvin, the bigness of a pease, lightly stamped, and drink it in the morning at your breaks-fast.^104^

The following excerpt found in Oxinden’s *Miscellany* appears to be a concise version of Vicary’s receipt: ‘The flower of Brimston, the yolke of an egge put into a wine glasse’.^105^ This exemplifies goal-orientated reading: Oxinden found a recipe for cough in his home library and, distilling it into its essential parts, noted it in his commonplace book for ready and accessible use in the future.

Oxinden’s annotations in his medical texts reveal other medical interests aside from his preoccupation with respiratory illness. He underlines sections in *Microkosmographia* (1631) relating to conception and the sex of the foetus.

That conception may be perfect, the seede which is yeelded and retained must be pure and fruitfull. By pure, I understand with Hippocrates, that which is not sickly or diseased, neither yet mingled with blood…

If after the third and fourth moneth the woman feele no motion the Conception is faulty; for sayeth Hippocrates, Male Infants do move the third moneth and Females the fourth. ^106^

Oxinden’s underlining suggests he was interested in requirements for a healthy conception and indicators of the health and gender of the foetus, attributed to Hippocrates. While studies often exclusively focus on the burden of childbearing on women, as Evans and Read argue, concerns regarding fertility, miscarriage and stillbirth also affected fathers.^107^ As the patriarch, Oxinden’s responsibility was to support his wife during pregnancy, nurture healthy offspring and ensure the continuation of the family line. Oxinden was fortunate to have a son and four daughters; the safe delivery of his children and the survival of a male heir was paramount. Oxinden’s interest in ‘faulty’ conception may indicate a concern regarding the pregnancies of either or both of his wives.

Oxinden was also attentive to the relatively new and highly contagious ‘French Disease’ (syphilis), described by the chemical physician, Marchamont Nedham, in *Medela Medicinae* (1665). Oxinden underlined the means of transmission, including ‘Carnal Contact’, ‘By ill cures’, ‘By accidental Contagion’, ‘By Hereditary Propagation’ and ‘By Lactation’.^108^ Regarding ‘carnal contact’, Oxinden underlined how the French Disease may go undetected for some time, presenting it as a hidden threat.

That after the committing of that Folly with an unwholsome Person, though there appear no Sign nor Symptom of a Disease for the present, yet it may be latent and lurking within the Body, many years, before it make any discovery of it self either in its own nature, or in the disguise of other Diseases. ^109^

In the case of gonorrhoea, also a sexually transmitted disease, young men who caught the infection from prostitutes were thought to infect their new brides, threatening the fertility of the marriage.^110^ Oxinden, aged 55 when given the book so not a young man himself, may have feared for his daughters’ risk of syphilitic infection from their husbands or the risk of his grandchildren being infected by their wetnurses.

Nedham also describes how the French disease can be transmitted accidentally and Oxinden drew a manicule in the margin to highlight this theory as well as underlining the text.

They touch one another; though not visibly, after the manner of Common Contact, yet every jot as effectually, by the intercourse of those Corpuscles which pass to and fro betwixt them. And thus, I suppose it is clear enough how the Pockie Lues may be propagated by accidental contagion even to innocent Persons. ^111^

This section explains the transmission of this devastating disease employing corpuscular philosophy, as particles move between bodies infecting even the ‘innocent’. Here, Oxinden showed interest in new theories of contagion as chemical physicians sought to understand epidemics beyond a Galenic model of explanation. Oxinden’s notes thus reveal a concern not only with the common afflictions of coughs and colds which troubled himself and his family but also deeper fears regarding problematic conceptions and pregnancies and the threat of new diseases spread by bodily fluids and ‘common contact’. These fears relate to the survival of future generations.

In contrast, the predominant concern of bachelor, Lee Warly, is related to more personal ailments: wind and digestion. Warly bought and annotated *The Anatomy of Melancholy* (1676); according to Burton, ‘windy or hypochondriacall melancholy’ could be caused inwardly by the spleen, belly or bowels or outwardly by abusing the six non-naturals.^112^ This outward type of melancholy was self-induced, caused by an imbalance in levels of fresh air, exercise, sleep, diet, excretion and passion. Warly enjoyed recording amusing rhymes on the subject. Inside the front cover, Warly wrote, ‘NB: The Advice of a Gent who died from neglecting it - Where e’er you be: Let farts go free’, underlining the comic phrase. He also copied out an excerpt from *Hudibras* (1663), the mock-heroic narrative poem by poet and satirist, Samuel Butler.

As wind I’ th Hypochondres pent

So but a Blast if downward sent

But if it upward chance to flie

Becomes new light and Prophecie.

-Butler’s Hudibras 2^nd^ part, canto 3^rd^.

If indeed it was he himself suffering from wind or a member of his household, Warly viewed the condition with humour. This would reflect Zwicker’s discussion of Jonathan Swift’s ‘fops’ at the turn of the eighteenth century who ‘revel in wit’ and mine for snippets of opinion.^113^ Yet elsewhere, Warly pursued practical cures. Later in *The Anatomy of Melancholy*, Warly marks lifestyle measures to combat wind with an ‘NB’: in the margin and underlining lifestyle advice such as ‘Put seven hours difference betwixt dinner and supper’, recommending that food must be ‘well chewed, and not hastily gobled’ and ‘to eat no more than he can well digest’.^114^ Inside the back cover of *Ars Churgica* by empiric, William Salmon, there is a scrawled annotation in Warly’s hand: ‘Page 677…Clyster, Wind’, referring to the Turpentine clyster receipt for ‘if the Wind is in the Guts’ on that page.^115^ As a rare reference to a specific medical recipe, it is intriguing to consider this as evidence that Warly was researching making remedies as well as purchasing them.

Melancholy was also associated with ageing and Warly underlines the relationship between the two. Warly was fortunate to live a long life, as did his mother who lived to the age of 87.

Old age, which being cold and dry, and of the same quality as melancholy is, must needs cause it, by diminuation of spirits and substance, and increasing of adult humours… After seventy years… all is trouble and sorrows…especially in such as have lived in action all their lives.^116^

As we have a sense that Mary and Lee Warly were both lively people who enjoyed witty repartee, this section suggests a concern with the ‘diminuation of spirits’ as the trials and tribulations of age inevitably caught up with them.

Warly’s interests appear to be rather self-absorbed, the preoccupations of a wealthy bachelor who feared the effects of eating too much, too fast and the perils of ageing. It is noteworthy that Burton’s text, first published in 1621 and reliant on Galenic medicine and diet, was a key source. In contrast, Oxinden had multiple concerns relating to his large family and gave credit to a range of sources, from ancient Hippocratic and Galenic explanations to newer theories in chemical medicine and natural philosophy.

As well as researching particular conditions relating to illness, reproduction and ageing, the notes and annotations of Oxinden and Warly demonstrate how they assessed the value of imported medical ingredients to domestic medicine. The English believed their bodies to be the finest in the world, tall, pale and strong, grown in the temperate Northern climate, far from the hot, putrid airs of the Mediterranean and the colonies. English health and strength depended on the diet, water, seasons and climate of home, an idea originating from the Hippocratic tradition of Airs, Water and Places: people’s physiology and character arise from their environment.^117^ Debates regarding whether imported foods and medicines were suitable for English constitutions raged in the seventeenth and eighteenth centuries amongst medics, theologians and authors of household manuals.^118^ While Oxinden sought to engage with new medicines within the familiar framework of Galenic medicine, Warly challenged the worth of medicines far beyond the realms of traditional English herbs.

In Oxinden’s copy of *Cosmographie* (1657), an immense tome giving the history and geography of the world, he underlined a poem by Alfred of Beverley about the British Isles.

A wealthy island, which no help desires,

Yet all the World supply for her requires

Able to glut King Solomon with pleasures

And further great Augustus with her treasures. ^119^

This annotation suggests that Oxinden appreciated imported materials as ‘treasures’ that the English had a right to enjoy and utilise. The inclusion of imported ingredients in at least 14 receipts in his *Miscellany* reflects a desire to exploit these ingredients from across the globe. One receipt for a cold and a cough demands an array of exotic ingredients including Italian plums and artichoke: ‘China roote heartes horne iuiubes alias iules, shauings of Ivory, oake of Ierusalem these boyled with a knuckle of veale to a gelly, take a porringer full boyle these from a pottle to a quart in a pipkin, take it at morning & at 4 of the Clocke’.^120^ Even one of Oxinden’s simpler remedies, a glass of brandy with sugar in it, demanded imported treasures.^121^ As Oxinden’s first wife, Anne, was frequently ill, Oxinden may have considered using these receipts for her, as the lady of the house.

While imported herbs with obvious effects could be subsumed within Galenic medicine, such as the purging qualities of senna, the propounded benefits of some imported ingredients recommended in medical receipts generated debate. Tobacco was a controversial substance, condemned by James I but imported in the seventeenth century in vast quantities from Virginia for both its pleasurable and curative properties.^122^ Oxinden explored tobacco as a potential treatment for his recurrent coughs. He has underlined ‘A large description of the hearbe Nicotiana or Petum’ on the contents page of *The Countrey Farme* (1616). Estienne writes a glowing account, ‘Nicotiana, though it haue beene but a while knowne in France, yet it holdeth the first and principall place amongst Physicke hearbes, by reason of his singular and almost diuine vertues’.^123^ He describes several miraculous cures of cancers, ulcers, eye complaints, poisoning and bites by mad dogs, attributed to tobacco. For the treatment of coughs, Estienne notes that tobacco is hot and dry and therefore suitable for cold, moist lung conditions, according to humoral theory: ‘Fume taken at the mouth is good for them that haue a short breath, old cough or rheumes, in which maketh them to auoyd infinite quantitie of thicke and slimie flegme’.^124^ However, Oxinden believed he had the wrong sort of cough to be treated with tobacco. He wrote ‘Dec 13, 1652, Mr. Alexander Rosse sayd Tobacco is naught for me. Hee sayd tobacco was dry and the lungs dry and that Coltsfoot and Anyseed is better.’^125^ In the front cover of his copy of *Medela Medicinae* (1665), Oxinden also noted, ‘Tobacco is most pernicious for me certainly, said A B Godson, Xmas day 1664.’^126^ Thus, although tobacco was described in wondrous terms, Oxinden did not accept it outright. The appeal of the exotic imported herb and its ‘divine vertues’ were trumped by the principles of Galenic medicine and in this case, familiar herbs more suited to his specific condition and constitution might be preferable.

Despite growing opportunities to travel in the eighteenth century, Warly was not a great traveller, dividing his time between Canterbury and the courts in London. He was also more ambivalent about the influx of imported ingredients than Oxinden. While keen to research imported ingredients, a trefoil next to an entry on the medical uses of China Root presents a discreet mark of interest, Warly’s concern regarding these imports is apparent in his annotations.^127^ Warly wrote out the following quote, likely copied from *Physico-Theology* (1742) by clergyman and natural philosopher, William Derham, where it is highlighted with a manicule: ‘Johannes Benovorinus a Physician of Dort wrote a Book on Purpose to show that every Country hath everything serving to its occasions and particularly Remedies afforded to all the Distempers it is subject unto.’^128^ Warly also copied it into the front pages of *A Treatise of Foods* (1704). This highlighting and copying may convey agreement with the statement. He also wrote out the following section from Virgil, *Georgics Liber 2*:

Oh happy swain, too happy if you knew

Your blest estate! Just earth prepares for you

Un-purchas’d food, far from wars dire debates.

Though no proud palaces, with lofty gates

Steam with the breath of Clients every morn;

Nor ivory, the carved posts adorn;

No brass of Corinth, rich embroidery,

No wool infected with Assyrian dye;

Nor Oyl with Cassia mixt: you gentle peace

Enjoy.^129^

This passage rejects exotic, expensive materials in favour of a bucolic vision of simple pleasures. The choice of the word ‘infected’ in this translation to describe using Assyrian dye and the rejection of Oil mixed with Cassia (Chinese Cinnamon) suggests that Warly too wished to express his rejection of extravagant, imported ingredients, in preference for the home-grown products of English rural life. There was also a strong association between foreign peoples and objects and disease transmission in the seventeenth and eighteenth centuries, with diseases such as syphilis identified as ‘French’.^130^ By using the term ‘infected’ Warly might imply that foreign dyes and spices were not only morally corrupt but physically contagious.

Warly applied this cautious approach to everyday life. In *The Compleat Housewife* (1758), Warly has drawn a manicule in the margin pointing to this line: ‘These receipts are all suitable to English Constitutions and English Palates’ and Smith recommends ‘such Provisions as are the Product of our Own Country’.^131^ Yet, Smith’s declarations are undermined by her section on French cookery, let alone the multitude of imported ingredients she lists in her medical receipts. A recommended ingredient might be found closer to home and Warly considered alternatives as demonstrated by his annotation in Chapter XLI on Saffron in Renou’s *A Medicinal Dispensatory* (1657).^132^ The author writes ‘the best of all grows in Corycus, a Mountain of Cilicia, for its odour is more fragrant and its colour more aureus’. Unsurprisingly, Warly was unimpressed and wrote in the margin, ‘As good, or better at Saffron Waldon in Essex in England’. Warly’s interest in saffron may have been related to its properties to aid digestion. In *The Anatomy of Melancholy* (1676), saffron is listed among simples as ‘correctors against wind, against costiveness etc’.^133^ If saffron could be sourced in Essex, more cheaply and conveniently, Warly was keen to consider this in preference to the luxurious and exotic saffron of the Mediterranean.

The cost of imported ingredients in comparison with local herbs was likely a consideration for Oxinden and Warly. Oxinden was financially burdened by long-running court cases and supporting his large family. Tobacco, sugar, spirits and wine, all used in home remedies, were subject to customs duties, increasing the costs.^134^ However, such medicines were clearly deemed a necessity, given the frequency of imported ingredients listed in Oxinden’s *Miscellany*. Oxinden recorded this instruction ‘Diacodium a spoonefull when one goes to bed tis 6d’ [t]he ounce Christoph Boyes’, attributed to ‘The lady Oxiden June 19, 1656’, likely his aunt Margaret.^135^ Christopher Boyes may have been the apothecary who set the price. Diacodium was a syrup of imported white poppies, with an opiate effect. At 96d per pound, this was extraordinarily expensive when compared to other simples, though evidently bought in very small quantities.^136^ While there were variations in price, the cost of some ingredients for medicinal use remained high in comparison to native English herbs.

While Warly expressed distaste for imported ingredients, we know that he bought expensive ‘sugars’ for his mother in London and it may be that expense was justified by necessity. The Warlys were certainly interested in frugality and self-reliance. This attitude could reflect the religious devotion and Protestant emphasis on restraint indicated by their book choices. Mary’s collection included *The Daily Self-Examinant* (4th edn, 1710), *The Practice of Piety* (60th edn, 1743) and *Pilgrim’s Progress* (3rd edn, 1690).^137^ Warly was mindful of needless extravagance and made his views on the matter known in comments in his account book: ‘Look about your houses, in every degree: And as your Gettings are, so let your Spendings be.’^138^ This rhyming couplet is probably not an original composition but suggests that the Warlys did not wish to consume beyond their means. He also writes, ‘In our Expenses we should neither ape those that are placed in a more exalted sphere, nor…sink beneath our proper station.’^139^ The reference to sanctity in his note ‘tis use that sanctifies expense’ highlights the religious aspect to spending, the sense that a pious and humble Protestant household morally justified expenditure only on the basis of necessity.^140^ Thus, the wealthy Warlys, who could afford expensive commodities, rejected the growing appetite for luxury during the eighteenth century.^141^

## Conclusion

Henry Oxinden and Lee Warly provide rich evidence for gentlemen’s interests in domestic medicine spanning the seventeenth and eighteenth centuries. Their circumstances were undoubtedly very different. Yet these gentlemen’s annotations, notes and letters reveal how knowledge was sought, shared and evaluated according both to practical need and academic interest, demonstrating a questioning, critical engagement with health information in a changing medical landscape.

Henry Oxinden faced the stresses of maintaining a large household and encountered frequent instances of ill-health during a turbulent period in English history. He required medical knowledge to fulfil his role as the patriarch and ensure the survival of his family. He sought the advice and reassurance of his aunt, consulted his network of contacts and his small selection of medical books, noting down information in his commonplace book and expressing his concerns in letters with family and friends. He drew on Hippocratic, Galenic and chemical frameworks to interpret health conditions and potential treatments. As an educated gentleman, Oxinden sought to maintain authority in terms of expertise within his household and participated in social networks which shared remedies and books.

Lee Warly, a wealthy bachelor who enjoyed a witty turn of phrase, could devote time, money and energy to amassing a large book collection and studying medicine as a gentlemanly pursuit in the relative stability of the eighteenth century. With medical knowledge and access to a wide medical marketplace, Warly felt confident to freely disparage physicians, express suspicion about imported medicines and search for advice and treatments from a range of sources. In caring for his mother, he was able to consult his medical books and buy medicines directly from apothecaries in London, judging their value to justify the expense.

These case studies demonstrate an enthusiasm and practical need for the gentlemanly study and sharing of medical knowledge and ambivalence regarding the merit of physicians and the worth of imported medicines. They also add nuance to our conceptualisation of gender roles within early modern domestic medicine, both in terms of study and practice. The absence of female marginalia in the family medical books offers a counterpoint to the scholarship on women’s active engagement with medical texts, asserting that while female book ownership and *reading* were encouraged, women’s *writing* in medical books was not always deemed necessary or desirable. These case studies also highlight the necessary fluidity of gender roles within the realities of family life. While women were expected to take primary responsibility for the practice of domestic medicine, female ill-health or incapacity due to the demands of pregnancy and child-birth or the frailty of old age likely placed a greater demand on men within the household than has previously been recognised.

Henry Oxinden and Lee Warly were thus ultimately responsible for the health and wellbeing of themselves and their households and they sought to exploit all avenues of knowledge available to them. Drawing on familial, social and professional networks and extensive libraries to develop their medical knowledge for practical as well as academic purposes, these practices also served to enhance their position as learned gentlemen. Analysis of their endeavours enriches the scholarship on English domestic medicine in the early modern period and presents opportunities for further research into the pursuit and application of domestic medical knowledge.

## Footnotes

1 Elaine Leong, ‘Collecting Knowledge for the Family: Recipes, Gender and Practical Knowledge in the Early Modern English Household’, *Centaurus*, 2013, 81−103; Roy Porter, ed, *Patients and Practitioners: Lay Perceptions of Medicine in Pre-industrial Society* (Cambridge: Cambridge University Press, 2002); Anne Stobart, *Household Medicine in Seventeenth Century England* (London: Bloomsbury, 2016); Alun Withey, *Physick and the Family: Health, Medicine and Care in Wales, 1600−1750* (Manchester: Manchester University Press, 2011).

2 Linda A. Pollock, *With Faith and Physic: The Life of a Tudor Gentlewoman, Lady Grace Mildmay, 1552− 1620* (Collins & Brown, 1993); Katherine Allen, ‘Hobby and Craft: Distilling Household Medicine in Eighteenth Century England’, *Early Modern Women: An Interdisciplinary Journal*, 2016, 11; Elaine Leong, *Recipes and Everyday Knowledge: Medicine, Science and the Household*, (Chicago: University of Chicago Press, 2018), see Lady Johanna St John, 46−70.

3 Gervase Markham, *The English Hus-wife* (London: 1615).

4 Roy Porter, ‘The Patient’s View: Doing Medical History from Below’, *Theory and Society*, 1985, 14, 175−98, 177, 180.

5 Lisa Smith, ‘The Relative Duties of a Man: Domestic Medicine in England and France, ca. 1685−1740’, *Journal of Family History*, 2006, 31, 237−56, 240; Hannah Newton, *The Sick Child in Early Modern England 1570−1720* (Oxford: Oxford University Press, 2012), 102−3, 120; Jennifer Evans and Sara Read, ‘“Before Midnight She Had Miscarried”: Women, Men, and Miscarriage in Early Modern England’, *Journal of Family History*, 2015, 40, 3−23, 15.

6 Smith, ‘Relative Duties’, 245.

7 Elaine Leong and Sara Pennell, ‘Recipe Collections and the Currency of Medical Knowledge in the Early Modern “Medical Marketplace”’, in *Medicine and the Market in England and Its Colonies C.1450−C.1850* (Basingstoke: Palgrave Macmillan, 2007), 133−52, 149.

8 Alun Withey, ‘Crossing the Boundaries: Domestic Recipe Collections in Early Modern Wales’, in *Reading and Writing Recipe Books, 1550−1800* (Manchester: Manchester University Press, 2013), 182.

9 Leong, ‘Collecting Knowledge for the Family’.

10 Henry Oxinden, *Miscellany of Henry Oxinden, ca. 1642−1670 V.b.110* (Washington DC: Folger Shakespeare Library); Dorothy Gardiner, ed, *The Oxinden Letters 1607−1642, Being the Correspondence of Henry Oxinden of Barham and His Circle* (London: Constable, 1933); Dorothy Gardiner, ed, *The Oxinden and Peyton Letters, 1642−1670: Being the Correspondence of Henry Oxinden of Barham, Sir Thomas Peyton of Knowlton and Their Circle* (London: Sheldon Press, 1937) (hereafter referred to as Gardiner, *Vol 1* and *Vol 2*).

11 Lee Warly bequeathed the collection to the Elham Parish; it was deposited in the Canterbury Cathedral Archive and Library in the 1930s. See Sarah Gray and Chris Baggs, ‘The English Parish Library: A Celebration of Diversity’, *Libraries & Culture*, 2000, 35, 414−33.

12 Jennifer Richards and Fred Schurink, ‘Introduction: The Textuality and Materiality of Reading in Early Modern England’, *The Huntington Library Quarterly*, 2010, 73, 345−61, 357.

13 Peter Murray Jones, ‘Book Ownership and the Lay Culture of Medicine in Tudor Cambridge’, in H. Marland, M. Pelling, eds, *The Task of Healing: Medicine, Religion and Gender in England and the Netherlands, 1450−1800* (Rotterdam: Erasmus, 1996), 49−68.

14 Kevin Sharpe and Steven N. Zwicker, ‘Introduction: Discovering the Renaissance Reader’ in K. Sharpe, S. N. Zwicker, eds, *Reading, Society and Politics in Early Modern England* (Cambridge, Cambridge University Press, 2003), 1−40, 25.

15 Lisa Jardine and Anthony Grafton, ‘“Studied for Action”: How Gabriel Harvey Read His Livy’, *Past & Present*, 1990, 129, 30−78, 30, Lisa Jardine and William Sherman, ‘Pragmatic Readers: Knowledge Transactions and Scholarly Services in Late Elizabethan England’, in A. Fletcher and P. Roberts, eds, *Religion, Culture and Society*, 102−24; Ann M. Blair, *Too Much to Know: Managing Scholarly Information Before the Modern Age* (New Haven: Yale University Press, 2010), 86.

16 Steven N. Zwicker, ‘The Constitution of Opinion and the Pacification of Reading’, in K. Sharpe and S. N. Zwicker, eds, *Reading, Society and Politics*, 295−316, 311.

17 Karen Harvey, ‘The History of Masculinity, circa 1650−1800’, *Journal of British Studies*, 2005, 44, 296−311.

18 Alexandra Shepard, ‘From Anxious Patriarchs to Refined Gentlemen? Manhood in Britain, Circa 1500−1700’, *J. Br. Stud*., 2005, 44, 281−95, 295.

19 Elaine Leong, ‘“Herbals She Peruseth”: Reading Medicine in Early Modern England’, *Renaissance Studies*, 2014, 28, 556−78.

20 *Shepard*, ‘Anxious Patriarchs’, 287.

21 Gardiner, *Vol 1*, xxx−xxxiii.

22 Oxinden, *Miscellany*, 8−13.

23 Nicholas Culpeper, *A Physical Directory: Or a Translation of the Dispensatory Made by the Colledge of Physitians of London*, 2nd edn (1650), ELHAM 129, Canterbury Cathedral Library, Canterbury, Kent (here-after all ELHAM catalogue numbers refer to collection held in Canterbury Cathedral Library); Thomas Nicols, *Gemmarius Fidelius, Or the Faithful Lapidary* (1659), ELHAM 623.

24 Charles Estienne, *Maison Rustique, or, The Countrey Farme, Richard Surflet (trans), corr. and enl. by Gervase Markham* (1616), ELHAM 98; Thomas Vicary, *The English Mans Treasure: With the True Anatomie of Mans Bodie* (1613), ELHAM 127; Helkiah Crooke, *Mikrokosmographia. A Description of the Body of Man*, 2nd edn (1631), ELHAM 123.

25 Marchamont Nedham, *Medela Medicinae* (1665), ELHAM 1245.

26 Peter Heylyn, *Cosmographie* (1657) ELHAM 59; Thomas Gataker, *Marriage Duties Briefely Couched Togither Out of Colossians, 3. 18, 19*, (1620), ELHAM 372(5); Francis Bacon, *The Essays, or counsels civill and morall* (1639), ELHAM 763.

27 Jardine and Grafton, ‘Studied for Action’, 30.

28 Sheila Hingley, ‘The Oxindens, Warlys and Elham Parish Library: A Family Library and Its Place in Print Culture in East Kent, (unpublished doctoral thesis, Canterbury Christ Church University, 2004), 70.

29 Karen Harvey, ‘The History of Masculinity’, 298, 306.

30 Helen Berry, ‘Polite Consumption: Shopping in Eighteenth-Century England’, *Transactions of the Royal Historical Society*, 2002, 12, 375−94, 385.

31 Louis Lémery, *A Treatise of Foods*, trans into English, (1704), ELHAM 744; Eliza Smith, *The Compleat Housewife Or, Accomplished Gentlewoman’s Companion*, 16th edn (1758), ELHAM 594.

32 Joannes Renodaeus, *A Medicinal Dispensatory*, *Richard Tomlinson (trans. and ed.)* (1657), ELHAM 130(2); John Garfield, ed, *A Physical Dictionary* (1657), ELHAM 130(1); John Berlu, *The Treasury of Drugs Unlock’d, Or a Full and True Description of All Sorts of Drugs and Chymical Preparations sold by Druggists*(1690), ELHAM 1268; *The Vermin-Killer, Being a Compleat and Necessary Family-Book*, (1754), ELHAM 1288.

33 Robert Burton, *The Anatomy of Melancholy*, 8th edn (1676), H/G-12-22, Canterbury Cathedral Library.

34 Hingley, ‘The Oxindens, Warlys and Elham Parish Library’, 233.

35 Nicholas Culpeper, *The English Physician Enlarged* (1698), ELHAM 1265.

36 Joseph Blagrave, *Supplement or Enlargement to Mr Nich Culpepper’s English Physitian*, (1674), ELHAM 691(1).

37 John Earle, *The World Displayed, Or, Several Essays*, *consisting of the Various Characters and Passions of Its Inhabitants* (1740) ELHAM 944.

38 William Derham, *Physico-theology, Or, a Demonstration of the Being and Attributes of God from his Works of Creation* (1742), ELHAM 344.

39 Jardine & Grafton, ‘Studied for Action’, 51.

40 Bruce T. Moran, ‘A Survey of Chemical Medicine in the 17th Century: Spanning Court, Classroom, and Cultures’, *Pharmacy in History*, 1996, 38, 121−33, 121−2.

41 Andrew Wear, *Knowledge and Practice in English Medicine, 1550−1680*, (Cambridge: Cambridge University Press, 2000), 419.

42 Mary Fissell, ‘Readers, Texts and Contexts: Vernacular Medical Works in Early Modern England’ in R. Porter, ed, *The Popularisation of Medicine 1650−1850* (Oxford: Routledge, 1992); Elizabeth Lane Furdell, *Publishing and Medicine in Early Modern England* (New York: University of Rochester Press, 2002).

43 Roy Porter, ‘Laymen, Doctors and Medical Knowledge in the Eighteenth Century: The Evidence of the Gentleman’s Magazine’, in R. Porter, ed, *Patients and Practitioners*, 283−314, 288; Sharon T. Strocchia, ‘Introduction: Women and Healthcare in Early Modern Europe’, *Renaissance Studies*, 2014, 28, 496−514, 513.

44 Leong and Pennell, ‘Recipes Collections’, 149.

45 Tessa Storey, ‘English and Italian Health Advice: Protestant and Catholic Bodies’, in Sandra Cavallo and Tessa Storey, eds, *Conserving Health in Early Modern Culture: Bodies and Environments in Italy and England* (Manchester: Manchester University Press, 2017), 227, 210−35.

46 Simon Werrett, *Thrifty Science: Making the Most of Materials in the History of Experiment* (Chicago: The University of Chicago Press, 2019), 36−8.

47 See Gardiner, *Vol 1*, 41, 122, 216, 249; *Vol 2*, 85.

48 Gardiner, *Vol 2*, 85.

49 Anthony Fletcher, ‘The Protestant Idea of Marriage in Early Modern England’, in A. Fletcher and P. Roberts, eds, *Religion, Culture and Society in Early Modern Britain*, (Cambridge: Cambridge University Press, 1994), 161−181, 174.

50 Gardiner, *Vol 1*, 131.

51 *Ibid*., 132.

52 *Ibid*., 179.

53 *Ibid*., 191−2.

54 Hingley, ‘The Oxindens, Warlys and Elham Parish Library’, 45.

55 Patrick Wallis, ‘Consumption, Retailing, and Medicine in Early-Modern London’, *The Economic History Review*, 2008, 61, 26−53, 31.

56 Cited in Hingley, ‘The Oxindens, Warlys and Elham Parish Library’, 106.

57 Claire Bartram, ‘The Reading and Writing Practices of the Kentish Gentry: The Emergence of a Protestant Identity in Elizabethan Kent’ (Unpublished doctoral thesis, University of Kent, 2005), 53.

58 Peter Laslett, ‘The Gentry of Kent in 1640’, *Cambridge Historical Journal*, 1948, 9, 148−64, 155; Joan Thirsk, The Fashioning of the Tudor-Stuart Gentry’, *Bulletin of the John Rylands Library*, 1990, 72, 69−86, 76−7.

59 Cited in Hingley, ‘The Oxindens, Warlys and Elham Parish Library’, Appendix 3.

60 For a similar collection of receipts from local acquaintances, see Fairfax family in Leong, *Collecting Knowledge*, 94.

61 Oxinden, *Miscellany*, 9−11.

62 Stobart, *Household Medicine*, 53.

63 Oxinden, *Miscellany*, 11.

64 Withey, ‘Crossing the Boundaries’, 195.

65 Ilana K Ben-Amos, *The Culture of Giving* (Cambridge: Cambridge University Press, 2008), 80.

66 Leong and Pennell, ‘Recipe Collections’, 133.

67 Hingley, ‘The Oxindens, Warlys and Elham Parish Library’, 70.

68 Ian Mortimer, ‘A Directory of Medical Personnel Qualified and Practising in the Diocese of Canterbury c. 1560−1730, Kent Archaeological Society’, *Archaeologica Cantiana*, 2006, 126, 135−70, 167.

69 Louise Hill Curth, ‘Medical Advertising in the Popular Press: Almanacs and the Growth of Proprietary Medicines’, in *From Physick to Pharmacology* (London: Ashgate, 2006), 29−48, 43.

70 David Allan, ‘Book-Collecting and Literature in Eighteenth-Century Britain’, *The Yearbook of English Studies*, 2015, 74−92, 82.

71 Edward H. Jacobs, ‘Buying Into Classes: The Practice of Book Selection in Eighteenth-Century Britain’, *Eighteenth-Century Studies*, 1999, 33, 43−64, 59.

72 Porter, ‘The Patient View’, 190, Furdell, *Publishing and Medicine*, 49−73.

73 Nedham, *Medela Medicinae*.

74 Hingley, ‘The Oxindens, Warlys and Elham Parish Library’, 36; See ‘Jacob, William: Physician of Canterbury’, in Mortimer, ‘A Directory’, 155.

75 This knowledge exchange is mentioned in the context of the eighteenth century by Roy Porter, in ‘Laymen, Doctors and Medical Knowledge’, 296.

76 Oxinden, *Miscellany*, 12.

77 Mortimer, ‘A Directory’,144; Gardiner, *Vol 2*, 204.

78 Oxinden, *Miscellany*, 8; Mortimer, ‘Fox, William’, in ‘A Directory of Medical Personnel’, 151.

79 Gardiner, *Vol 1*, 131; See ‘Randal/Randolph, Edmund’, in Mortimer, ‘A Directory’, 161.

80 Gardiner, *Vol 1*, 179.

81 Harold J. Cook, ‘Good Advice and Little Medicine: The Professional Authority of Early Modern English Physician’, *J. Br. Stud*., 1994, 33, 1−31, 4.

82 Nedham, *Medela Medicinae*, 422−3.

83 ’The English Iliads, A Poem by the Rev John Warly MA and late Fellow of Clare Hall in Cambridge, printed in the year 1674’ in the front page of Lémery, *A Treatise of Foods*.

84 Garfield, *Physical Dictionary*, A4.

85 *Ibid*., B1.

86 Earle, *World Display’d*, 12−13.

87 Storey, ‘English and Italian Health Advice’, 220.

88 Herman Boerhaave (1668−1738), *Nature*, 1938, 142, 504.

89 Porter, ‘Laymen, Doctors and Medical Knowledge’, 296 and ‘The Patient View’, 189.

90 Wear, *Knowledge & Practice*, 472.

91 Culpeper, *English Physician Enlarged*, 454−56.

92 See Leong, *Recipes*, 49, and ‘Herbals She Peruseth’; Stobart, *Household Medicine*.

93 *The Gentlewoman’s Companion: Or A guide to the Female Sex* (1678), 183−4.

94 Gataker, *Marriage Duties*, 17.

95 Fletcher, ‘Protestant Marriage’, 170.

96 Sanders, Eve, *Gender and Literacy on Stage in Early Modern England*, (Cambridge: Cambridge University Press, 1999), 170; Carol Winkelmann, ‘A Case Study of Women’s Literacy in the Early Seventeenth Century: The Oxinden Family Letters’, *Women and Language*, 1996, 19.

97 Heidi Brayman Hackel, ‘“Boasting of Silence”: Women Readers in a Patriarchal State’, in K. Sharpe and S. N. Zwickers, eds, *Reading, Society and Politics*, 101−21, 107.

98 Nathaniel Spinckes, *The True Church of England’s Man’s Companion in the Closet* (1712) ELHAM 694.

99 Brayman Hackel, ‘Boasting of Silence’, 110.

100 Winkelmann, ‘A Case Study’, 3−5.

101 Bacon, *The Essays*, of Studies, 293−4.

102 Burton, *Anatomy of Melancholy*. 434.

103 Cited in Hingley, ‘The Oxindens, Warlys and Elham Parish Library’, 45.

104 Vicary, *English Man’s Treasure*, 210.

105 Oxinden, *Miscellany*, 10.

106 Crooke, *Microkosmographia*, 297−8.

107 Evans and Read, ‘Before Midnight’, 13, 16.

108 Nedham, *Medela Medicinae*, 62.

109 *Ibid*., 63.

110 Smith, ‘Relative Duties’, 248.

111 Nedham, *Medela Medicinae*, 130.

112 Burton, *Anatomy of Melancholy*, 256−8.

113 Zwicker, ‘The Constitution of Opinion’, 296.

114 Burton, *Anatomy of Melancholy*, 149.

115 William Salmon, *Ars Churgica*, Vol 1, *ELHAM 1251, 677*.

116 Burton, *Anatomy of Melancholy*, 36.

117 Andrew Wear, ‘Place, Health, and Disease: The Airs, Waters, Places Tradition in Early Modern England and North America’, *Journal of Medieval and Early Modern Studies*, 2008, 38, 443−65.

118 Stobart, *Household Medicine*, 95; Wear, *Knowledge and Practice*, 76.

119 Heylyn, *Cosmographie*, 293.

120 Oxinden, *Miscellany*, 10.

121 *Ibid*.

122 Ghislaine Lawrence, ‘Tobacco Smoke Enemas’, *The Lancet*, 2002, 359, 1442−5, 1443.

123 Estienne, *Countrey Farme*, 215.

124 *Ibid*., 220.

125 Oxinden, *Miscellany*, 9.

126 Nedham, *Medela Medicinae*.

127 Garfield, *Physical Dictionary*, 6.

128 Derham, *Physico-Theology*, 422.

129 Estienne, *Countrey Farme*, inside front cover.

130 Nedham, *Medela Medicinae*, 62.

131 Smith, *Compleat Housewife*, A3−4.

132 Renodaeus, *Medicinal Dispensatory*, 251.

133 Burton, *Anatomy of Melancholy*, 249.

134 Stobart, *Household Medicine*, 85−6.

135 Oxinden, *MIscellany*, 8.

136 Stobart, *Household Medicine*, 85−6.

137 Robert Warren, *The Daily Self-Examinant: Or an Earnest Perswasive to the Duty of Daily Self-Examination*, 4th edn (1710), ELHAM 1230; Lewis Bayly, *The Practice of Pietie*, 60th edn (1743), ELHAM 341; John Bunyan, *The Pilgrim’s Progress* (1693), ELHAM 1083.

138 Lee Warly, ‘Cash Account Book of Lee WARLY of Blean, Near Canterbury, Co. Kent’, (1766) KEN 14/2/1, Museum of English Rural Life, University of Reading, Reading, Berkshire, 5.

139 Warly, ‘Cash Account Book’, 1.

140 Warly, ‘Cash Account Book’, back flyleaf.

141 John Brewer and Roy Porter, eds, *Consumption and the World of Goods* (London: Routledge, 1997); Lorna Weatherill, *Consumer Behaviour and Material Culture in Britain, 1660−1760* (London: Routledge, 1996).

